# Supplementary material for: Prediction Model for Pre-Eclampsia Using Gestational-Age-Specific Serum Creatinine Distribution
Source: Biology (Basel). 2023 Jun 4;12(6):816. doi: 10.3390/biology12060816 (PMC10295363; doi:10.3390/biology12060816)
Supplement: Supplementary file 1 [file biology-12-00816-s001.zip › biology-2382825-supplementary.pdf]

Supplementary Materials

Prediction Model for Pre-Ecalmpsia Using Gestational Age Specific Serum Creatinine

Jieun Kang [1], Sang Won Hwang [2], Taesic Lee [3], Jooyoung Cho [4], Dong Min Seo [5], Seong Jin Choi [1,*], Young Uh [4,*]

1 Department of Obstetrics and Gynecology, Yonsei University Wonju College of Medicine, Wonju 26411, Republic of Korea
2 Artificial Intelligence Bigdata Medical Center, Yonsei University Wonju College of Medicine, Wonju 26411, Republic of Korea
3 Department of Family Medicine, Yonsei University Wonju College of Medicine, Wonju 26411, Republic of Korea
4 Department of Laboratory Medicine, Yonsei University Wonju College of Medicine, Wonju 26411, Republic of Korea
5 Department of Medical Information, Yonsei University Wonju College of Medicine, Wonju 26411, Republic of Korea

* Correspondence: choisj@yonsei.ac.kr; u931018@yonsei.ac.kr; Tel.: +82-33-741-1592

1. *Definitions of adverse pregnancy outcomes*

PE was defined as the presence of HTN and proteinuria occurring after 20 weeks of gestation in a previously normotensive patient, based on the American College of Obstetrics and Gynecology [17,18].

PTB was determined as birth before 37 completed weeks of gestation. This definition is recommended by the World Health Organization and is used in the International Classification of Diseases. Infants born toward the end of this preterm period were traditionally considered to have "lower risk" [19]. Therefore, babies born between 34+0 weeks and 36+6 weeks of GA were defined as late preterm infants. Moreover, PTB was subdivided into early PTB (~ 33+6 weeks) and late PTB (34+0 to 36+6 weeks).

FGR was defined as a failure to achieve the appropriate growth potential. Fetuses with birth weight below the 10th percentile are at an increased risk of stillbirth or perinatal mortality, while those with birth weight below the 3rd percentile have the highest risk. At present, there is no gold standard for the diagnosis of FGR. Therefore, it is usually defined as the statistical deviation of the fetal size from a population-based reference, typically at thresholds in the 10th, 5th, or 3rd centile and most commonly in the 10th centile [20,21].

Serum Creatinine data

id	date	mt	cr	GAQ
[redacted]	2010[redacted]	0.29	79.560	0
[redacted]	2010[redacted]	0.14	79.560	0
[redacted]	2012[redacted]	0.43	44.200	0
[redacted]	2011[redacted]	0.14	61.880	0
[redacted]	2011[redacted]	0.14	61.880	0
[redacted]	2014[redacted]	0.29	61.880	0
[redacted]	2014[redacted]	0.86	50.388	0
[redacted]	2016[redacted]	0.29	66.300	0
[redacted]	2010[redacted]	0.14	53.040	0
[redacted]	2013[redacted]	0.71	67.184	0
[redacted]	2016[redacted]	0.14	63.648	0
[redacted]	2012[redacted]	0.43	57.460	0
[redacted]	2013[redacted]	0.14	62.764	0
[redacted]	2011[redacted]	0.57	61.880	0
[redacted]	2019[redacted]	0.29	60.996	0
[redacted]	2011[redacted]	0.57	53.040	0
[redacted]	2011[redacted]	0.86	61.880	0
[redacted]	2015[redacted]	0.29	65.416	0
[redacted]	2018[redacted]	0.43	60.996	0
[redacted]	2013[redacted]	0.86	67.184	0

Anthropometric data

id	wt	ht	bmi
[redacted]	106.00	162.0	40.39018
[redacted]	67.60	160.7	26.17670
[redacted]	80.20	170.0	27.75087
[redacted]	76.00	169.3	26.51549
[redacted]	76.00	167.0	27.25089
[redacted]	62.00	160.0	24.21875
[redacted]	47.80	151.0	20.96399
[redacted]	60.50	162.5	22.91124
[redacted]	59.00	162.0	22.48133
[redacted]	62.45	168.7	21.94332
[redacted]	63.60	163.0	23.93767
[redacted]	61.00	156.0	25.06575
[redacted]	76.00	162.0	28.95900
[redacted]	80.00	162.0	30.48316
[redacted]	61.80	165.0	22.69972
[redacted]	108.00	176.1	34.82612
[redacted]	57.00	163.0	21.45357
[redacted]	75.00	163.0	28.22839
[redacted]	90.00	165.0	33.05785
[redacted]	47.00	153.0	20.07775

Lab data

patient.no	alp	ast	alt	ldh	bun	ggt	test.date
[redacted]	96	15	9		4	16	201[redacted]
[redacted]	147	77	59		5	28	201[redacted]
[redacted]		57	31	379	26.8		201[redacted]
[redacted]	75	33	15		14	7	201[redacted]
[redacted]		15	6			12	201[redacted]
[redacted]	59	22	22		14.2	15	202[redacted]
[redacted]	114	58	30	292	8.9	71	202[redacted]
[redacted]	73	21	8		11.3	11	201[redacted]
[redacted]	62	20	12		17	5	201[redacted]
[redacted]		20	< 6	283	12.5		201[redacted]
[redacted]		18	11	221	8.1		201[redacted]
[redacted]	61	17	26	202	11.1	28	201[redacted]

Figure S1. Data structure

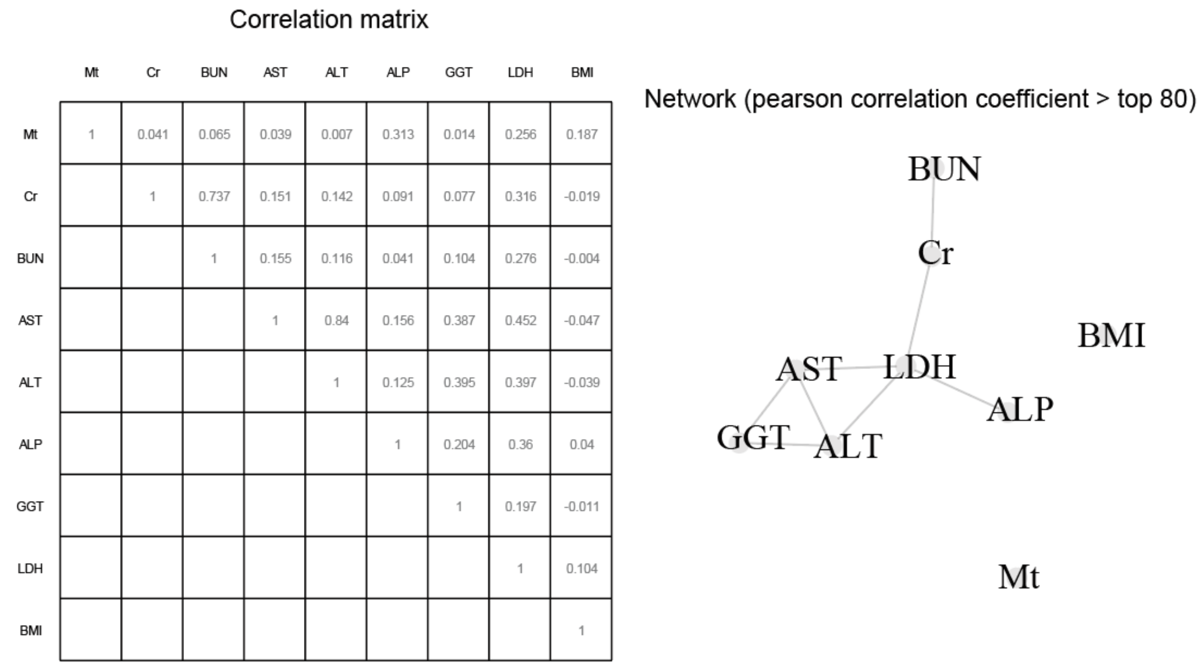

Figure S2. Correlation analysis among candidate markers.

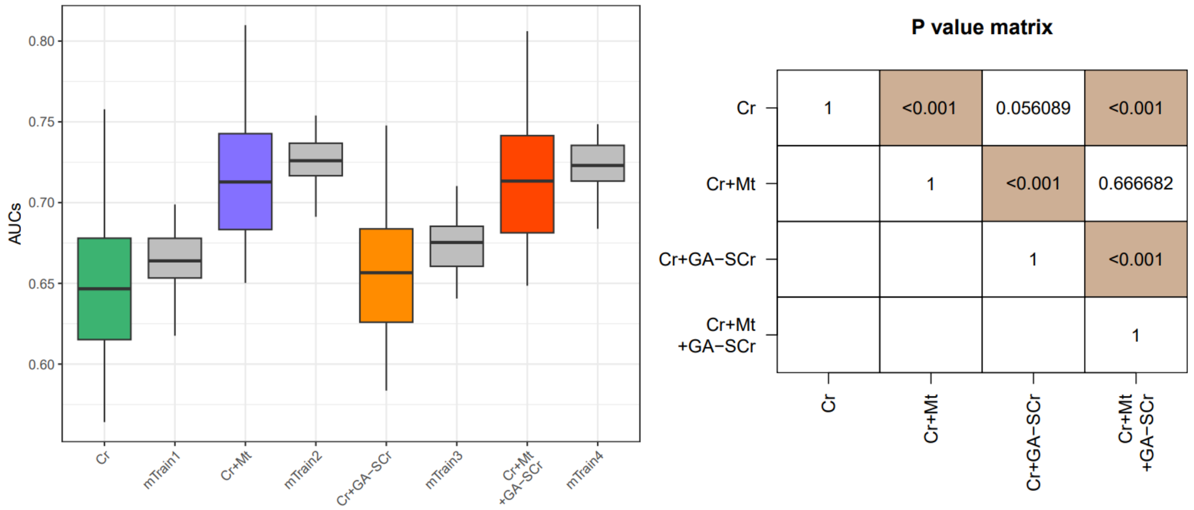

Figure S3. Prediction performances of PE with late PTB using the four feature-sets. Any cases of PE (PE only + PE with other adverse pregnancy outcomes) were determined as the disease group. Prediction model was established using logistic regression. Boxplots include minimum, first quartile (25%), median, third quartile, and maximum values. Points in the boxplot referred to as outliers indicate cases showing more than 1.5 times the interquartile range (IQR), biased from the matched median value. Y-axis indicates AUC for predicting PE, and the box plot summarizes 100 levels of AUCs. Green, purple, orange, and red colored boxplots were obtained from the testing dataset while grey colored boxplots are curated from the training dataset. Abbreviations: PE, preeclampsia; SCr, serum creatinine; Mt, measured time; GAQ, gestational age-specific SCr quartile; IQR, interquartile range; AUC, area under receiver characteristic curve.

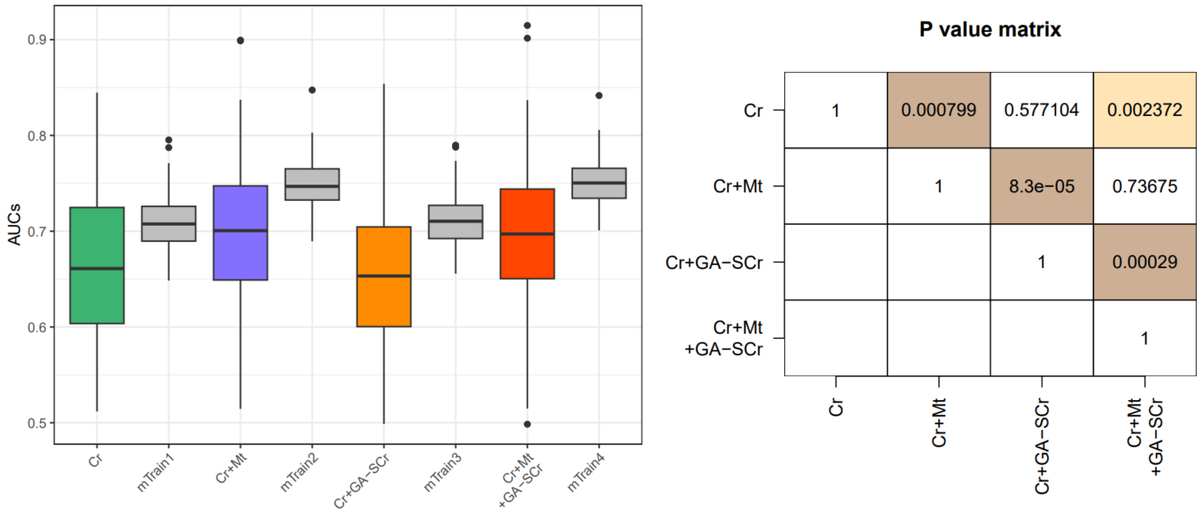

Figure S4. Prediction performances of PE with FGR using the four feature-sets. Any cases of PE (PE only + PE with other adverse pregnancy outcomes) were determined as the disease group. Prediction model was established using logistic regression. Boxplots include minimum, first quartile (25%), median, third quartile, and maximum values. Points in the boxplot referred to as outliers indicate cases showing more than 1.5 times the interquartile range (IQR), biased from the matched median value. Y-axis indicates AUC for predicting PE, and the box plot summarizes 100 levels of AUCs. Green, purple, orange, and red colored boxplots were obtained from the testing dataset while grey colored boxplots are curated from the training dataset. Abbreviations: PE, preeclampsia; SCr, serum creatinine; Mt, measured time; GAQ, gestational age-specific SCr quartile; IQR, interquartile range; AUC, area under receiver characteristic curve.

Table S1. General participant characteristics according to the GAQ.

	GAQ1	GAQ2	GAQ3	GAQ4	P for trend
N	5847	1794	1517	968	
Age	33.17 ± 0.07	33.65 ± 0.11	33.56 ± 0.13	33.6 ± 0.16	<0.001
Labor types, n					
Nullipara	2595 (44.4)	696 (38.8)	552 (36.4)	362 (37.4)	<0.001
Multipara	3252 (55.6)	1098 (61.2)	965 (63.6)	606 (62.6)	<0.001
Essential hypertension, n	72 (1.2)	17 (0.9)	31 (2)	33 (3.4)	<0.001
Diabetes, n	188 (3.2)	75 (4.2)	54 (3.6)	90 (9.3)	<0.001
PE, n	461 (7.9)	221 (12.3)	243 (16)	291 (30.1)	<0.001
PE alone, n	75 (1.3)	37 (2.1)	40 (2.6)	17 (1.8)	0.001
PE+FGR, n	37 (0.6)	25 (1.4)	19 (1.3)	33 (3.4)	<0.001
PE+PTB, n	151 (2.6)	79 (4.4)	75 (4.9)	127 (13.1)	<0.001
PE+FGR+PTB, n	198 (3.4)	80 (4.5)	109 (7.2)	114 (11.8)	<0.001
SCr, µmol/L	40.35 ± 0.11	53.01 ± 0.13	60.11 ± 0.17	116.18 ± 3.21	<0.001
SCr < 30	575	0	0	0	
30 ≤ SCr <40	2109	0	0	0	
40 ≤ SCr <50	2537	567	2	0	
50 ≤ SCr <60	600	942	799	14	
60 ≤ SCr <110	26	285	716	718	
eGFR ≥ 110	0	0	0	236	

Continuous and categorical variables are presented as mean ± SD and number (percent), respectively. Abbreviations: SCr, serum creatinine; SD, standard deviation; PE, preeclampsia; FGR, fetal growth retardation; PTB, preterm birth.
